# Deriving accurate microbiota profiles from human samples with low bacterial content through post-sequencing processing of Illumina MiSeq data

**DOI:** 10.1186/s40168-015-0083-8

**Published:** 2015-05-05

**Authors:** Jake Jervis-Bardy, Lex E X Leong, Shashikanth Marri, Renee J Smith, Jocelyn M Choo, Heidi C Smith-Vaughan, Elizabeth Nosworthy, Peter S Morris, Stephen O’Leary, Geraint B Rogers, Robyn L Marsh

**Affiliations:** Menzies School of Health Research, Child Health Division, Charles Darwin University, Darwin, NT Australia; School of Medicine, Flinders University, Bedford Park, Adelaide, SA Australia; Infection and Immunity Theme, South Australia Health and Medical Research Institute, North Terrace, Adelaide, SA Australia; School of Biological Sciences, Flinders University, Adelaide, South Australia 5001 Australia; Department of Otolaryngology, University of Melbourne, Melbourne, VIC Australia

**Keywords:** 16S rRNA, Respiratory, MiSeq, Contamination, Pair-end reads, QIIME, Otitis media

## Abstract

**Background:**

The rapid expansion of 16S rRNA gene sequencing in challenging clinical contexts has resulted in a growing body of literature of variable quality. To a large extent, this is due to a failure to address spurious signal that is characteristic of samples with low levels of bacteria and high levels of non-bacterial DNA. We have developed a workflow based on the paired-end read Illumina MiSeq-based approach, which enables significant improvement in data quality, post-sequencing. We demonstrate the efficacy of this methodology through its application to paediatric upper-respiratory samples from several anatomical sites.

**Results:**

A workflow for processing sequence data was developed based on commonly available tools. Data generated from different sample types showed a marked variation in levels of non-bacterial signal and ‘contaminant’ bacterial reads. Significant differences in the ability of reference databases to accurately assign identity to operational taxonomic units (OTU) were observed. Three OTU-picking strategies were trialled as follows: *de novo*, open-reference and closed-reference, with open-reference performing substantially better. Relative abundance of OTUs identified as potential reagent contamination showed a strong inverse correlation with amplicon concentration allowing their objective removal. The removal of the spurious signal showed the greatest improvement in sample types typically containing low levels of bacteria and high levels of human DNA. A substantial impact of pre-filtering data and spurious signal removal was demonstrated by principal coordinate and co-occurrence analysis. For example, analysis of taxon co-occurrence in adenoid swab and middle ear fluid samples indicated that failure to remove the spurious signal resulted in the inclusion of six out of eleven bacterial genera that accounted for 80% of similarity between the sample types.

**Conclusions:**

The application of the presented workflow to a set of challenging clinical samples demonstrates its utility in removing the spurious signal from the dataset, allowing clinical insight to be derived from what would otherwise be highly misleading output. While other approaches could potentially achieve similar improvements, the methodology employed here represents an accessible means to exclude the signal from contamination and other artefacts.

**Electronic supplementary material:**

The online version of this article (doi:10.1186/s40168-015-0083-8) contains supplementary material, which is available to authorized users.

## Background

The development of high-throughput, low-cost, sequencing has greatly expanded the ability of researchers to investigate complex bacterial systems associated with the human body. In particular, 16S rRNA gene amplicon sequencing has been used widely, most commonly in the characterisation of samples from ‘high biomass’ sites such as the gastrointestinal tract. Samples from such contexts are comparable in richness and complexity to some of the environmental microbial systems for which high-throughput sequencing technology was pioneered, allowing the technology to be applied with relatively minor modifications. However, amplicon- sequencing approaches are also being applied increasingly to anatomical [1,2] and environmental sites [3] that contain very low levels of bacteria such as the distal airways in the absence of an infection [1,2]. Here, a number of factors can have a major impact on the data generated. These include a reduction in PCR amplification efficiency due to high levels of human nucleic acids and low levels of bacterial 16S rRNA gene copies that result in increased sampling bias. Of particular concern is the contribution of low levels of signal from non-bacterial DNA and bacterial DNA present as reagent contamination [4], which would not substantially affect sequencing data in samples containing high concentrations of bacterial template. Such a spurious signal can substantially distort community profiles from samples with low bacterial load [4-6]. When 16S rRNA gene sequencing is applied to such contexts without due consideration of these factors, it can give rise to a conclusion that is potentially misleading [4,7]. The impact of failing to perform necessary data processing steps to render data accurate and clinically informative can be particularly problematic in studies that rely on commercial-sequencing providers. The absence of these steps, which are not standard for commercial-sequencing firms, has resulted in an increasing body of literature, whose quality is highly variable [4]. Nevertheless, some well-conducted studies have attempted to address these issues, providing clinically robust conclusions [8].

Our aim was to develop a methodology that allows non-specialist researchers to derive accurate and clinically informative data from Illumina MiSeq-based pair-end 16S rRNA gene profiles generated from challenging respiratory contexts. Rather than distinguishing between a genuine signal and a spurious signal based on subjective ‘balance of probability’ assessments, our approach is based on defined parameters that can be applied objectively and uniformly. Further, it was our intention that, wherever possible, this methodology would be based on commonly available software, with a minimal requirement for specialist bioinformatic expertise. By applying our methodology to a collection of nasopharyngeal (NP) swabs, adenoid biopsies, adenoid swabs and middle ear fluid (MEF) samples from indigenous Australian children with otitis media with effusion (OME, the presence of middle ear fluid behind an intact tympanic membrane without signs or symptoms of infection), we illustrate the beneficial impact of this workflow on bacterial community data from a challenging clinical context.

## Results and discussion

The total number of sequences successfully assembled from paired-end reads across the sample set was 2,094,672. Following quality filtering, truncation and chimera removal, a total number of 1,706,072 sequences advanced to operational taxonomic unit (OTU) picking and taxonomy assignment.

### Reference database selection for OTU picking and taxonomy assignment

**Figure 1:**
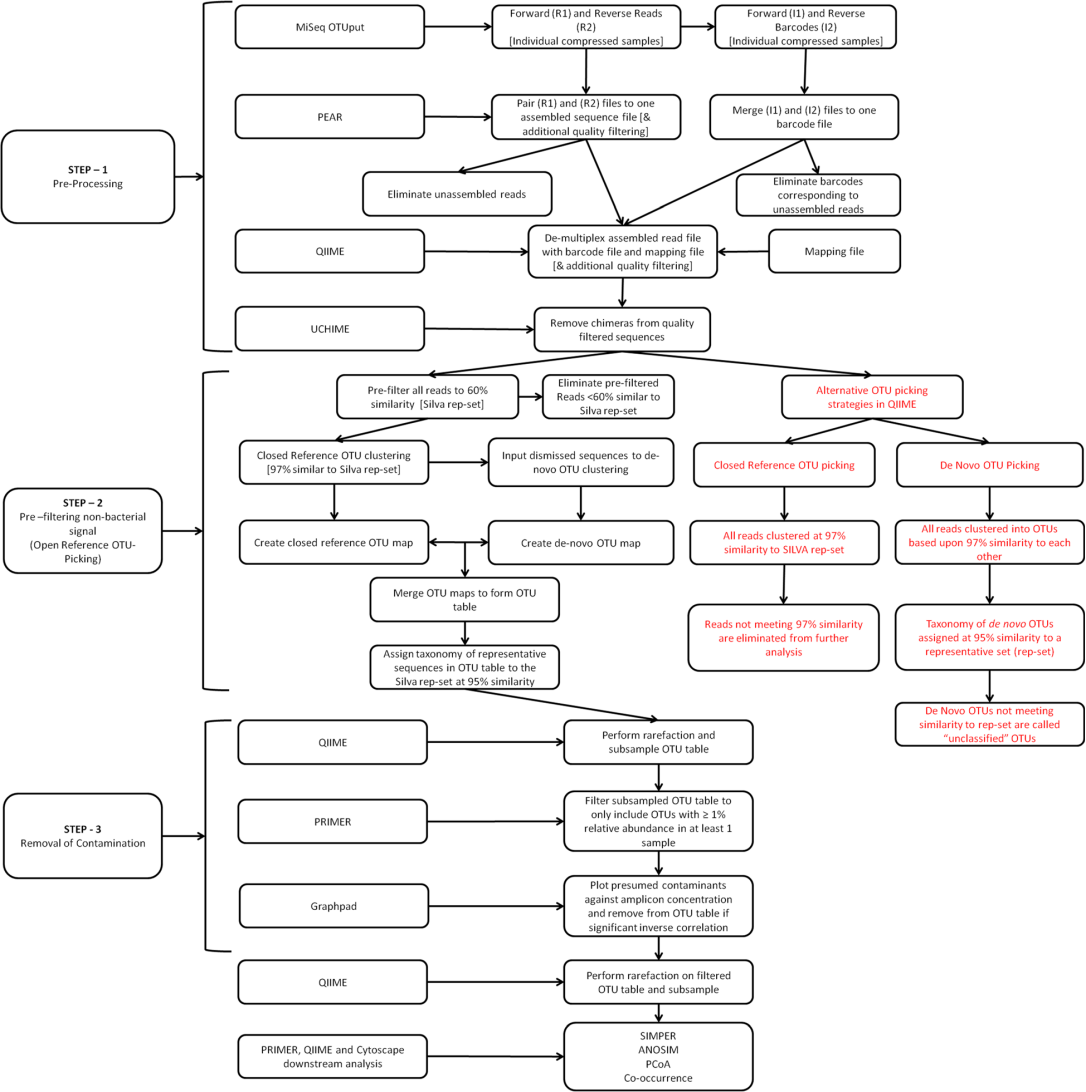
Workflow of bioinformatic and biostatistical analysis.

A number of different databases of 16S rRNA gene sequences can be used to assign taxonomic identities to OTUs (Figure 1, Step 2). We compared the ability of two of the most popular databases, Greengenes (v13.8) [9] and SILVA (v111) [10], to assign identities to OTUs generated from a multi-template control comprised of known species at defined relative abundance. These two databases were found to perform quite differently (Additional file 1: Tables S2, S3 and S4). Using identical OTU picking and taxonomic assignment methods, the Greengenes representative set did not assign taxonomy to eight of the top seventeen most abundant OTUs in the multi-template control (MTC). Importantly, Greengenes failed to assign *Moraxella* or *Staphylococcus* (common upper-airway colonisers) to any of the top 17 OTUs. In contrast, the SILVA representative dataset only failed to assign taxonomy to five of the top seventeen OTUs (and none of the top ten) and successfully assigned taxonomy to the most abundant *Moraxella* (17.6% relative abundance) and *Staphylococcus* OTUs observed in the MTC. Based on this analysis, SILVA was selected as the reference database for OTU picking and taxonomic assignment.

### Selection of OTU picking and taxonomic assignment strategy

We investigated three OTU picking approaches available in quantitative insights into microbial ecology (QIIME) as follows: *de novo* OTU picking, open-reference and closed-reference OTU picking (Figure 1, Step 2). Classical *de novo* OTU clustering and taxonomic assignment resulted in 108,099 individual OTUs clustered at 97% similarity with the majority of these OTUs ‘unclassified’ according to the representative set of sequences. Using sequence classification tool Kraken v0.10.5, 61.27% of all reads in the dataset were found to be non-bacterial sequences aligned to the human genome GRcH38 (Additional file 1: Figure S2) [11]. The *de novo* strategy was unable to eliminate these reads as a non-bacterial signal and instead classified the reads as unclassified. Using open-reference OTU picking, the percentage of the unclassified reads decreased to 2.5% with only 2,096 OTUs identified. The open-reference OTU picking method successfully eliminated the non-bacterial human signal associated with the *de novo* strategy while retaining unclassified bacterial reads. For example, retained unclassified bacterial reads such as *Alloiococcus* were >60% similar (the pre-filter cutoff) and <95% similar (the taxonomic assignment cutoff) to the SILVA database. Closed-reference OTU picking performed poorly as a number of high relative abundance OTUs were unclassified and eliminated from the final-output OTU table. In closed-reference OTU picking, all reads <97% similar to the SILVA database were discarded, meaning taxa such as *Alloiococcus* were unclassified and eliminated from the dataset, despite the open-reference method returning a cumulative relative abundance of 42.7% for *Alloiococcus* in the MEF samples. Accordingly, the open-reference method for OTU picking was employed for all further analyses.

### Impact of pre-filtering on adenoid sample types

**Figure 2:**
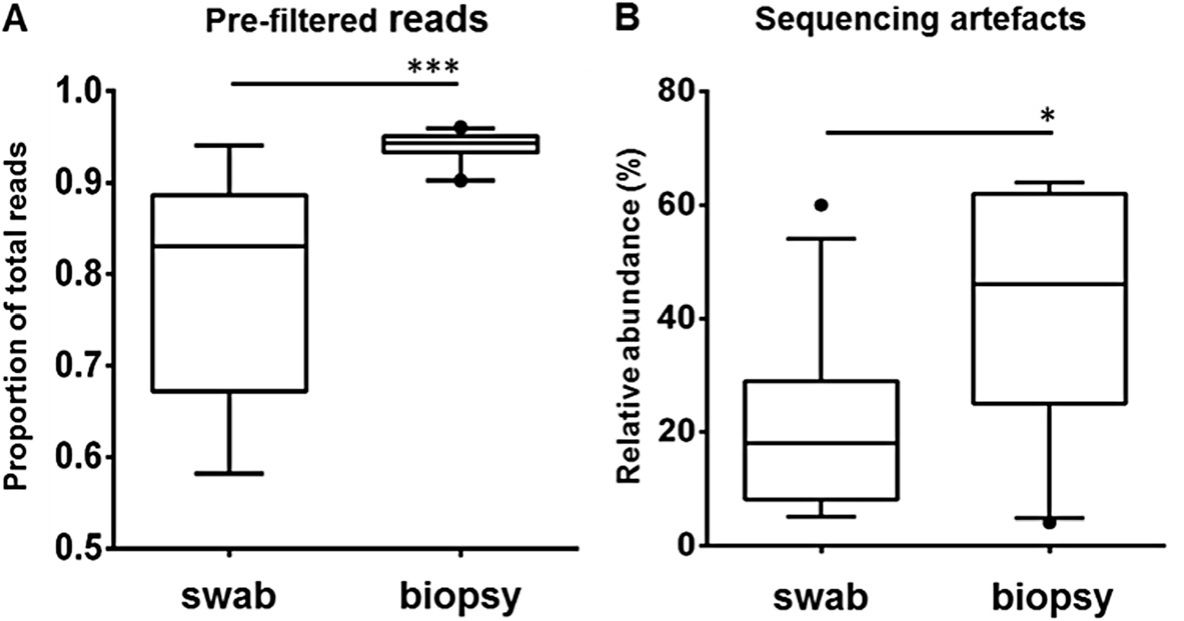
Proportion of sequence reads removed from adenoid swab compared to adenoid biopsy. **(A)** Proportion of reads removed by pre-filtering human OTUs in adenoid swab (*n* = 11) and adenoid biopsies (*n* = 11). ***P < 0.002, Mann–Whitney test. **(B)** Relative abundance of sequences identified as artefact in adenoid swab and adenoid biopsies. *P < 0.032, Mann–Whitney test.

The impact of the pre-filtering step in open-reference OTU picking, which eliminated the non-bacterial signal, was demonstrated by the analysis of the differences observed across the adenoid specimen sample types. A significantly higher percentage of reads in each sample were removed by pre-filtering from the adenoid biopsies (93.8%, SD 0.02), which contain high levels of human material relative to bacterial content, compared to the adenoid swabs (78.4%, SD 0.13) (*t*-test, *P* = 0.002) (Figure 2A). As discussed above, aligning sequences to the human genome (GRcH38) indicated that these reads were derived from human DNA. Where a high percentage of the total reads in the biopsies were human, the resulting yield of bacterial sequences was significantly lower, with a median of 947 (IQR 2507) reads in the adenoid swabs and 94 reads (IQR 76) in adenoid biopsies (Mann–Whitney *P* < 0.0001). The disparity in bacterial reads obtained from the adenoid swabs compared to the biopsies strongly suggest reduced 16S rRNA gene amplification efficiency, most likely due to competitive or inhibitory interactions arising from the high levels of non-bacterial DNA in the adenoid biopsies. We note there are a variety of stand-alone methods available to eliminate this type of non-bacterial signal, such as DeconSeq [12], Kraken [11] and FastQ Screen (Babraham Bioinformatics, Babraham Institute, UK). However, by utilising parameters in QIIME, we were able to achieve an efficient removal of the non-bacterial signal within the QIIME pipeline.

### Identification and removal of contaminants based on OTU distribution relative to biomass

**Figure 3:**
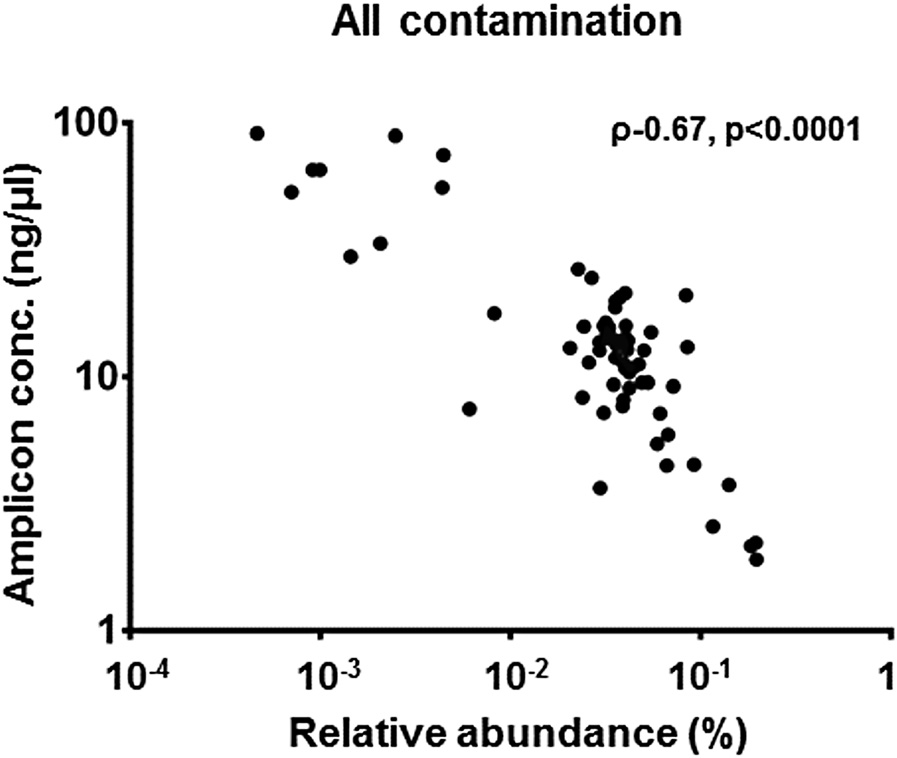
Relative abundance of OTUs identified as potential sequencing artefact plotted against amplicon concentration following library preparation. Spearman’s rho (ρ) and significance of correlation are shown.

**Figure 4:**
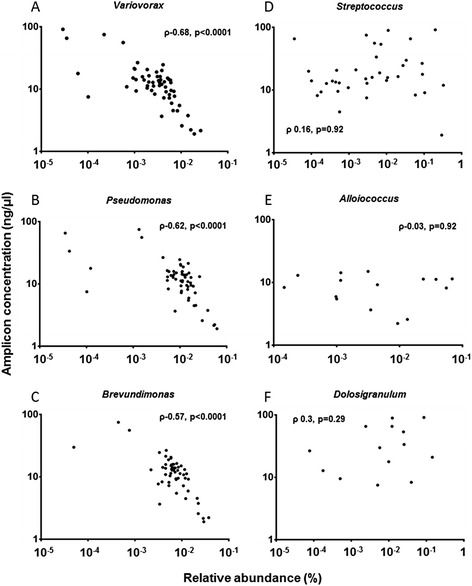
Relative abundance of presumed contamination and genuine signal plotted against amplicon concentration. Relative abundance of OTUs of sequencing artefacts **(A-C)** and non-artefacts **(D-F)** plotted against amplicon concentration following library preparation. Spearman’s rho (ρ) and significance of correlation are shown.

Following pre-filtering, the second stage of the pipeline involved the removal of presumed contaminants (Figure 1, Step 3). The relative abundance of all OTUs identified as potential reagent contamination showed a strong inverse correlation with amplicon concentration after 16S library preparation (*R* = −0.64, *P* < 0.0001, Spearman’s correlation) (Figure 3). This significant inverse relationship was also demonstrated at the individual OTU level (Figure 4A, B, and C). In contrast, OTUs representing genera thought not to be reagent contaminants showed no such correlation (Figure 4D, E and F). Such a relationship has been reported previously, based on 454 sequencing data [6]. OTUs were therefore removed on an objective basis, where a significant Spearman’s correlation (*P* ≤ 0.05) between amplicon concentration and OTU relative abundance was observed.

The relative abundance of reagent contaminants was significantly higher in the adenoid biopsies (median = 46%, IQR 37%) compared to the adenoid swabs (median = 18%, IQR 21%, *P* = 0.032, Mann–Whitney *U* test) (Figure 2B), consistent with our observation that the biopsies had reduced bacterial amplification efficiency. In addition, MEF samples also had a high relative abundance of reagent contaminants (median = 26%, IQR 24%). The high relative abundance of reagent contaminants in the MEF samples was also in the context of low biomass, with only eight out of twenty-two MEF samples successfully amplifying above the limit of detection in the total bacterial load qPCR (bacterial load in these swabs ranged from 1.7 × 10^4^ to 9.6 × 10^4^ copies ml^−1^ of MEF). By comparison, NP swabs had a contamination median relative abundance of only 0.2% (IQR 1.1%). Not surprisingly, this was in the context of the highest observed bacterial loads, with ten out of eleven swabs amplifying successfully (bacterial load in these swabs ranged from 2.7 × 10^4^ to 5.9 × 10^6^ copies ml^−1^ of swab and a mean of 22,431 non-contaminant bacterial sequences (SD 9,276)). Nasal cavity and NP swabs are the mainstay of upper-respiratory microbiome studies [5] and were less subject to the effect of non-bacterial DNA and reagent contaminants that we have observed in the other sample types (MEF and adenoid biopsy samples). The NP swabs therefore provided us with a baseline to assess how other lower biomass sample types behave using identical laboratory and bioinformatic methods.

It is important that the removal of contaminant taxa is performed at the OTU level. A number of genera known to be common colonisers of the upper-respiratory tract *(Stenotrophomonas* and *Pseudomonas)* have been identified as common reagent contaminants. Analysis of the distribution of these taxa at a genus level, where multiple OTUs are included in one correlation plot, could be misleading if some OTUs within that genus are spurious and others are not. Further, if researchers are concerned that there may be a mixture of genuine and contaminating reads within a single OTU clustered at 97% similarity, further analysis within that OTU could be performed. Differentiation might, for example, be achieved by clustering OTUs at a similarity of 100% and plotting each of the resulting OTUs against amplicon concentration. Ultimately, such removal of contaminants based on the relationship between OTU distribution and amplicon concentration may be fully automatable.

**Figure 5:**
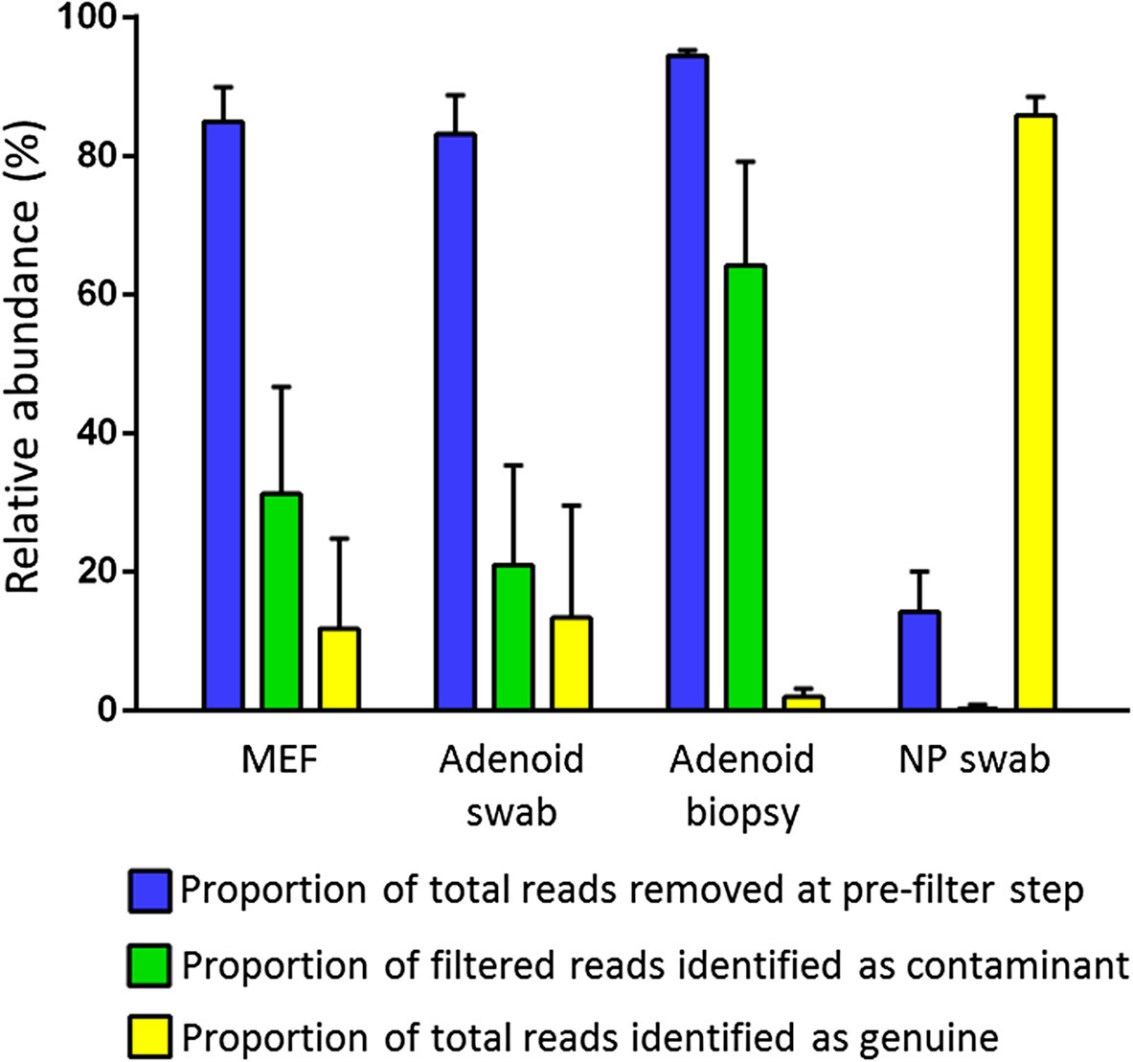
Proportion of sequence reads removed by pre-filtration or when identified as contaminants. The proportion of the total number of sequence reads obtained that were removed at the pre-filtering step or after being identified as potential contamination are shown for each of the four sample types. Also shown, the proportion of total sequence reads that were representative of the bacterial signal. Proportions do not sum up to 100% in each sample type as the reads removed as contaminants are shown as a proportion of the filtered reads.

The contribution of sequence reads that were removed at the pre-filtering step, removed after being identified as reagent contamination, or that were representative of presumed genuine bacterial signal are summarised for each of the four sample types in Figure 5. In the absence of pre-filtering and reagent contaminant removal, median relative abundance of presumed genuine bacterial signal in the NP swabs was above 85%, suggesting that potentially meaningful results from the NP swabs may have been achieved using other methods. Adenoid swabs and MEFs, however, contained low levels of bacteria and high levels of human DNA, with a median relative abundance of presumed bacterial signal lower than 15%. In these circumstances, an overwhelming spurious signal made it nearly impossible to derive accurate 16S data without pre-filtering and removing potential contamination.

### Impact of potential reagent contamination on measures of microbiota similarity

**Figure 6:**
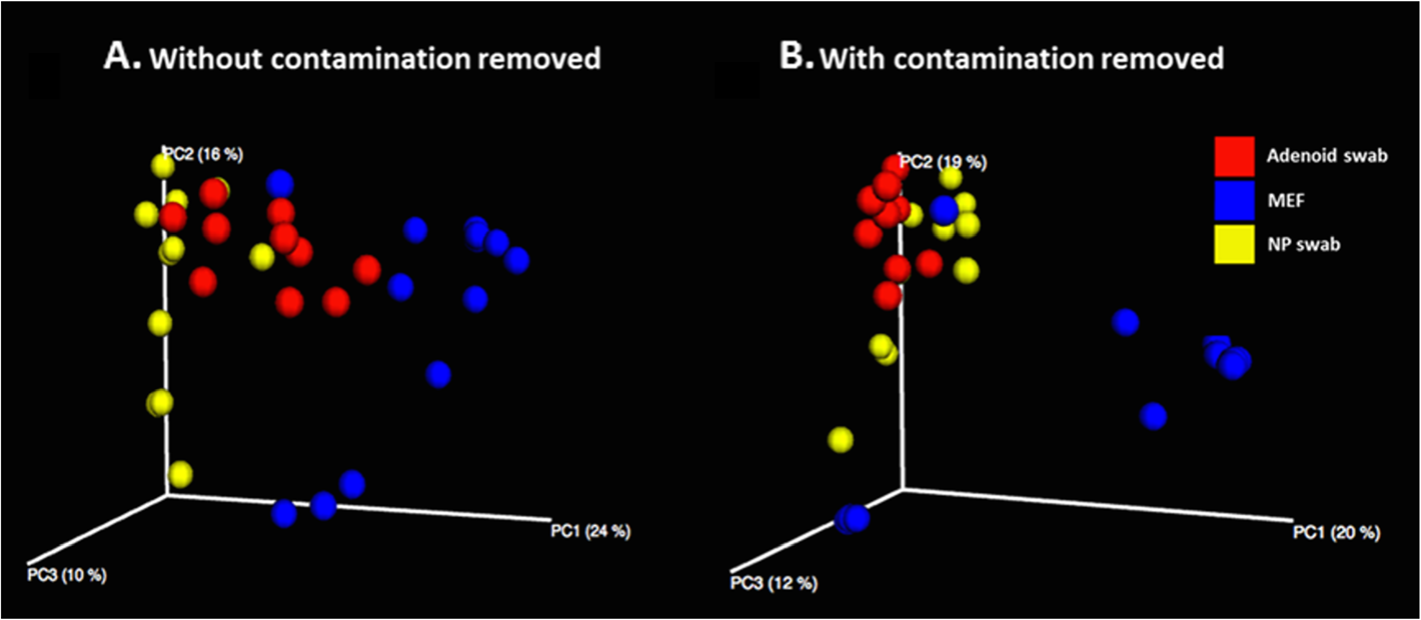
Principal coordinate plots based on a BC similarity matrix of bacterial community sequence data. Distribution of adenoid swabs, middle ear fluid and nasopharyngeal swabs are shown before **(A)** and after **(B)** removal of signal derived from contaminants. Panel A is based on 11 adenoid swabs, 13 middle ear fluid and 11 N swabs, with panel B based on 10 adenoid swabs, 11 middle ear fluid and 11 nasopharyngeal swabs.

To assess the impact of reagent contaminants in the dataset, we performed principal coordinate analysis (PCoA) based on sequence data before and after their removal (Figure 6A and B). Following the removal of contaminant OTUs, the NP and adenoid swabs showed a more proximal distribution. In contrast, the separation between MEFs and the other two sample sites increased. Analysis of similarity (ANOSIM) tests were used to determine whether differences between the distributions of the microbiota profiles from the three samples sites were significant. Both filtered and non-filtered datasets showed highly significant differences between the MEF and upper-airway samples (*P* < 0.001). The *R*-statistic for the filtered data set was 0.66 compared to 0.48 for the non-filtered data indicating that MEF samples were more dissimilar from the upper-airway samples following the removal of contaminant OTUs.

**Figure 7:**
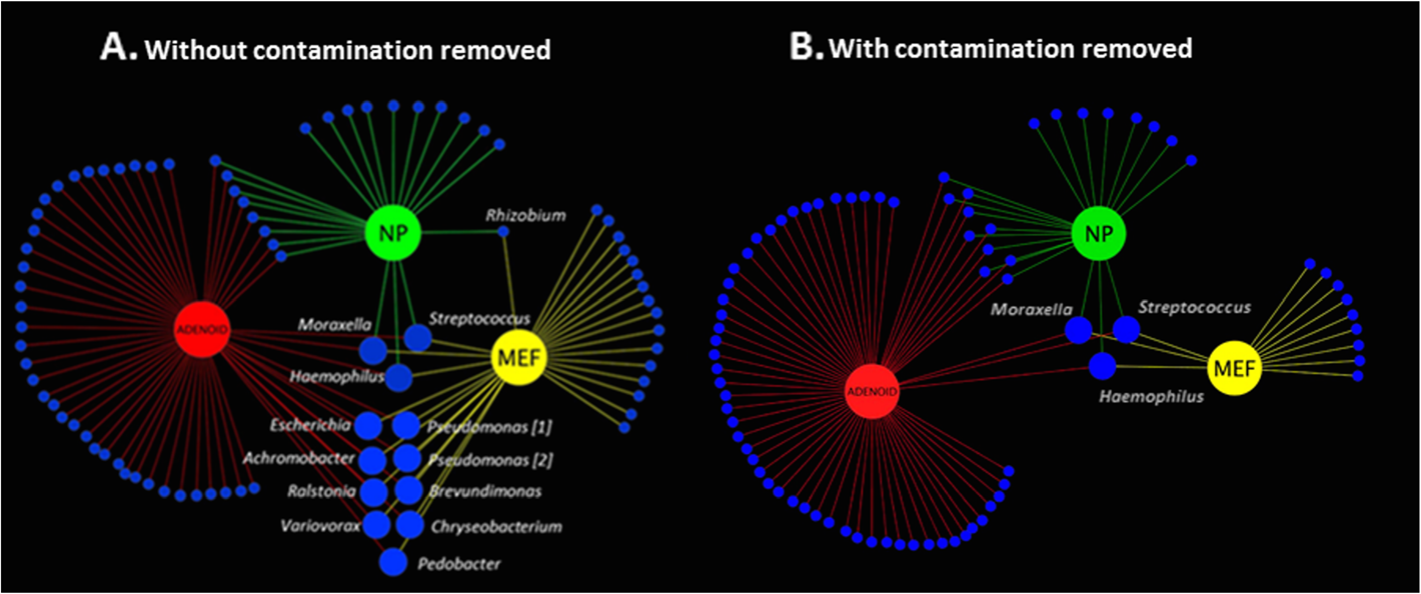
Co-occurrence plots for genera detected in adenoid swabs, middle ear fluid, and nasopharyngeal swabs. Plots are shown for data before **(A)** and after **(B)** removal of the signal derived from contaminants. Genera detected in both adenoid and MEF are highlighted. Note that *Achromobacter* was not removed based upon spurious distribution. However, following the removal of all contaminant OTUs and rarefaction to 400 reads, the sample with *Achromobacter* at >1.5% relative abundance was removed due to low sequencing depth. The removal based on spurious distribution of a *Rhizobium* OTU, detected in both NP and MEF, is also shown.

The impact of OTUs filtered from the dataset upon interpretation was further illustrated by an analysis of co-occurrence. The occurrence and co-occurrence of taxa present in each of the three sample types are shown based on non-filtered and filtered data (Figure 7A and B, respectively). To illustrate disparity, we highlight the co-occurrence of taxa in the adenoid swab and MEF, where 12 taxa are present at both sites when filtering is not performed, but this is reduced to three common colonisers of the upper airways with filtering (*Moraxella*, *Haemophilus* and *Streptococcus*). Taxa removed included *Ralstonia*, *Variovorx*, *Escherichia*, *Brevundimonas*, *Chryseobacterium*, *Pedobacter* and *Pseudomonas*. This observation was confirmed by similarity of percentage (SIMPER) analysis, where six out of the eleven OTUs contributing to 80% of the similarity between the MEF and adenoid swabs were identified as presumed reagent contaminants. Failure to identify these contaminants in the context of comparing the microbiome of the adenoids and MEFs in children with otitis media has the potential to produce highly misleading clinical findings. As the nasopharynx and adenoids are considered to be the reservoir of microbiota causing otitis media [13]; any organisms identified in adenoid and MEF samples would ordinarily be considered of potential clinical importance in the pathogenesis of this condition. However, as shown in our analysis, six out of eleven of the OTUs contributing to 80% of the similarity between the adenoids and MEFs were reagent contaminants.

## Conclusions

Currently, there is considerable interest in understanding the relationships between complex airway microbiota, host physiology and the development and progression of disease. In many cases, the low cost of high-throughput amplicon sequencing provide an opportunity to researchers and clinicians to perform such studies. However, while these technologies are commonly accessible, their application alone is not sufficient to provide informative data, particularly where airway samples contain low levels of microbes and/or high levels of human DNA. Careful bioinformatic processing is required to minimise the substantial impact of the spurious signal and to avoid basing clinical interpretation on potentially misleading output [14].

We describe a workflow for the removal of the spurious signal that can be applied using commonly available tools and without the need for highly specialised bioinformatics expertise. This approach could be easily applied to non-human studies and is not necessarily specific to 16S rRNA studies, as metagenomic approaches encounter similar issues [15]. To assess the efficacy of this approach, we applied it to the processing of microbiota data from human respiratory samples using largely standard protocols for DNA extraction, library preparation and sequencing. Specifically, we analysed sample types that contained different levels of bacterial and non-bacterial DNA. While our results suggest that for some sample types such as NP swabs, reasonably high quality data can be obtained without the need for stringent processing; the overwhelming artefact signal in the low bacterial biomass samples (MEF, adenoid swabs and adenoid biopsies) means that comparisons of microbial communities across all sample types is not possible in its absence. Further, parallel analysis of bacterial community distributions, with and without the removal of this signal, clearly indicated that failure to apply this methodology would have resulted in data that were misleading, pointing as it did to greater communication between anatomical sites than was indeed the case.

It is important to note that the level of the spurious signal within the sequencing data will be influenced by a wide range of factors and may differ substantially with sampling strategies, anatomical sites and even between replicate samples. Further, the fact that reagent contamination can be almost impossible to exclude entirely and the importance of using PCR primers that are able to amplify sequences from as great a proportion of bacterial species as possible, mean that the likelihood of preventing non-specific amplification is very low. As such, the development of an error-free method for generating 16S rRNA sequence data from clinical samples would seem highly unlikely. Methods that are able to remove spurious signal post-sequencing are therefore important protocol adjuncts. While we do not suggest that the methodology described here is definitive or that other approaches could not achieve similar results, we feel that our workflow provides a means for bench-top biologists with minimal bioinformatics experience to process data from challenging clinical contexts.

## Methods

Samples were collected as part of the National Health and Medical Research Council-funded (Grant 1007641) randomised controlled trial of surgical interventions for OME (Australia and New Zealand Clinical Trials Registration 12611001073998). Samples were collected at baseline from children undergoing surgery at the Alice Springs Hospital during May and June 2014. Ethical approval was obtained in the Northern Territory through the Central Australian Human Research Ethics Committee (HOMER 12 to 16) and Menzies School of Health Research Ethics Committee (2011 to 1686).

### Surgical procedures and sample collection

The clinical samples included 11 nasopharyngeal (NP) swabs, 22 middle ear fluid samples (MEFs) in saline (left and right ear of each child) and 11 adenoid biopsies. Swabs of the exterior of the adenoid biopsies were taken prior to DNA extraction. Full details of the surgical procedures and sample collection protocols are provided in Additional file 1.

### DNA extraction and estimation of total bacterial load

The total DNA was extracted from all clinical samples and two DNA extraction reagent negative controls. Full details of the DNA extraction protocols are provided in Additional file 1. Total bacterial load was determined as described previously [16] and was used to assess template concentrations for 16S rRNA amplicon sequencing. Full details of the qPCR protocol are provided in Additional file 1. A multi-template control (MTC) consisting of thirteen species in known relative abundance was used in the assessment of OTU picking and taxonomic assignment protocols. Full details of this MTC are given in Additional file 1: Table S1.

### 16S rRNA gene amplicon library preparation and sequencing

Amplicons were generated using fusion degenerate primers 27 F (5’-*TCGTCGGCAGCGTCAGATGTGTATAAGAGACAG*AGRGTTTGATCMTGGCTCAG-3’) and 519R (5’-*GTCTCGTGGGCTCGGAGATGTGTATAAGAGACAG*GTNTTACNGCGGCKGCTG-3’) with ligated overhang Illumina adapter consensus sequences in italic text. Full details of the library preparation and sequencing protocol are provided in Additional file 1. In brief, the initial PCR reactions were performed on a Veriti 96-well Thermal Cycler (Life Technologies, Australia). The PCR reactions were performed in the following programme: initiation enzyme activation at 95°C for 3 min, followed by 25 cycles consisting of denaturation at 95°C for 30 sec, annealing at 55°C for 30 sec and extension at 72°C for 30 sec. After 25 cycles, the reaction was completed with a final extension of 7 min at 72°C.

The Illumina Nextera XT Index kit (Illumina Inc., San Diego. CA, USA) with dual 8-base indices were used to allow for multiplexing. Two unique indices located on either end of the amplicon were chosen based on the Nextera dual-indexing strategy. To incorporate the indices to the 16S amplicons, PCR reactions were performed on a Veriti 96-well Thermal Cycler (Life Technologies, Australia). Cycling conditions consisted of one cycle of 95°C for 3 min, followed by eight cycles of 95°C for 30 sec, 55°C for 30 sec and 72°C for 30 sec, followed by a final extension cycle of 72°C for 5 min.

Prior to library pooling, the barcoded libraries were quantified using the Qubit dsDNA HS Assay Kit (Life Technologies, Carlsbad, CA, USA). Results from this quantification step (amplicon concentration) were used in downstream processing to eliminate contamination (Figure 1, Step 3). The libraries were sequenced by 2 × 300 bp paired-end sequencing on the MiSeq platform using MiSeq v3 Reagent Kit (Illumina) at the Flinders Genomics Facility, Adelaide, Australia. All sequence data generated have been submitted to the Sequence Read Archive [17].

### Bioinformatic processing

An overview of the bioinformatic workflow used is shown in Figure 1. FastQC v.11.2 (Babraham Bioinformatics, Babraham Institute, UK) was used to analyse the average quality scores of each sample before and after pairing reads. The Paired-End reAd mergeR (PEAR) v.0.9.5 [18] was used to pair the forward and reverse reads of sequences in each sample and discard all sequences less than 450 bp and/or with a Phred score <33. Kraken v0.10.5 [11] was used to classify sequences against pre-built databases of viral and bacterial sequences and the human genome (GRcH38). The pre-built databases (MiniKraken) were downloaded from the Kraken website (https://ccb.jhu.edu/software/kraken/ accessed 03032015), and query sequences were classified using Kraken’s default parameters.

Samples were then demultiplexed using QIIME v.1.8.0, with individual sequences assigned to their original samples. The demultiplex step contained further quality filtering steps as follows: truncation following three consecutive low quality base calls, removal of reads with <75% high quality base calls and removal of sequences with an unclear base call (N). Chimeras were filtered with a reference-based approach using UCHIME v.4.2 [19] and a representative set of chimera-checked sequences (Greengenes v.13.8; [9]).

For OTU picking, we used QIIME [20] as opposed to other popular 16S data analysis pipelines such as mothur [21]. We found the clustering mechanism employed by mothur version 1.34.3 unsuitable for processing paired-end read sequence data. In brief, the mothur MiSeq standard operating procedure relies on an OTU-clustering mechanism (nearest, furthest or average neighbour clustering) that generates a distance matrix, optimised when large data sets are condensed into a small number of identical sequences using the unique.seqs command. MiSeq sequencing generates paired-end reads with the potential for errors in the paired region of the sequences as previously described [18]. Consequently, when applied to large Illumina data sets, the unique.seqs command may be unable to condense data to an appropriate size for the distance matrix, resulting in excessive wall time during clustering. QIIME provides various OTU-clustering approaches (including those employed by mothur), some of which do not require generation of a large distance matrix (for example, UCLUST [22]).

Traditional *de novo* OTU picking, closed-reference and open-reference OTU picking were performed in QIIME. In *de novo* OTU picking, all reads were clustered based upon 97% similarity to each other, irrespective of similarity to known 16S rRNA sequences [23]. Taxonomy of *de novo* OTUs was assigned at 95% similarity to a representative set (rep-set) of 16S rRNA sequences in the SILVA database (release 111, July 2013) [10]). In closed-reference OTU picking, all reads were clustered based upon 97% similarity to a reference sequence in the rep-set, with all unassigned sequences discarded.

In open-reference OTU picking [24], all sequences were initially pre-filtered to discard sequences not meeting a threshold of 60% similarity to the rep-set. A closed-reference OTU-picking step was then performed, where all reads were clustered based upon 97% similarity to the rep-set. Reads failing to meet the 97% similarity threshold, were then clustered *de novo* (described above). OTU maps created for the closed-reference and *de novo* steps were then merged to create a combined OTU map. A representative set of sequences was created from the combined OTU map and taxonomy was assigned as described above. In the *de novo*, closed- and open-reference approaches, UCLUST [22] v.1.2.22 was used to cluster OTUs at 97% similarity. Analysis of a multi-template control was used to assess suitability of 16S rRNA reference databases for the taxonomic assignment. Taxonomic assignment for all three OTU picking methods was performed at 95% similarity to the rep-set using UCLUST.

### Biostatistical analysis

All samples were filtered to retain OTUs with a relative abundance of ≥1% in at least one sample. This allowed for manual inspection of individual OTUs for potential reagent contaminants. OTUs previously reported as common artefacts in sequence data from low biomass clinical samples were identified from the filtered OTU table [4]. The relative abundance of these OTUs was compared to the amplicon concentration measured during library preparation across all samples. OTUs were filtered from the data set if the distribution was inversely correlated with amplicon concentration, suggestive of a spurious signal generated in the absence of a preferentially amplified template [6]. In addition, OTUs identified in the DNA extraction negative controls were filtered from the sequence data. A complete list of OTUs removed in these steps is provided in Table 1, with sequences corresponding to OTU identities included in Additional file 2.

**Table 1:** **OTUs removed from sequencing data prior to biostatical analysis**

**Genus**	**OTU identity**
*Achromobacter*	JF925009
*Acidovorax*	JN869209, HQ681993
*Bergeyella*	New. CleanUp. ReferenceOTU46637
*Brevundimonas* ^a^	EF600592
*Candidatus Planktoluna*	FN668204
*Cellulosimicrobium*	New. CleanUp. ReferenceOTU1526
*Chryseobacterium* ^a^	New. ReferenceOTU87, New. CleanUp. ReferenceOTU40460, New. CleanUp. ReferenceOTU30994, AY46848
*Clavibacter*	New. ReferenceOTU27
*Devosia*	AY162048
*Flavobacterium*	New. ReferenceOTU91, New. CleanUp. ReferenceOTU22231
*Gelidibacter*	New. CleanUp. ReferenceOTU10780
*Janthinobacterium*	EU801443
*Mesorhizobium*	DQ228360
*Ochrobactrum*	DQ860022
*Pedobacter*	New. CleanUp. ReferenceOTU91
*Pelomonas* ^a^	JF733429, FJ269077
*Phyllobacterium* ^a^	GQ255500
*Pseudomonas* ^a^	JN187532, JF970596, GU272272, FJ347714, EF515711
*Ralstonia* ^a^	GU940710
*Rhizobium*	GQ472936
*Rhodanobacter*	New. CleanUp. ReferenceOTU3920
*Sphingomonas*	EF098188
*Stenotrophomonas*	FJ184356, AY373393
*Terrimonas*	New. CleanUp. ReferenceOTU20538
*Turicibacter*	AY953239
*Undibacterium*	GU940681
*Variovorax* ^a^	GU731299, GU272259

^a^Indicates contaminant OTUs detected in the DNA extraction negative controls. Sequences corresponding to OTU identities are included in Additional file 2 (filtered_contaminants.fna). Where accession numbers are given for OTU identities, the representative sequence from our dataset is 97% similar to the actual sequencing pertaining to that accession number.

OTUs that were not classified below family-level by taxonomic assignment based on the rep-set were further classified to obtain a genus- and species-level identification. This was achieved by aligning representative sequences of each selected OTU to the 16S ribosomal RNA sequence database and National Center for Biotechnology Information (NCBI) database using the Basic Local Alignment Search Tool (BLAST) [25]. Presumptive identification was made if an aligned sequence returned an identification coverage score of ≥97%. Where representative sequences aligned to multiple species with an identical coverage score ≥97%, a higher-level taxonomic identifier was assigned.

Rarefaction curves were generated in QIIME for all contaminant-filtered and non-filtered samples. Appropriate subsample depth was established by visual inspection of rarefaction curves to ensure adequate sample depth while retaining low read samples. It was confirmed that reducing the sequence number in this way did not result in a significant reduction in profile diversity, as determined using the Simpson’s Index of Diversity (1-D) (Additional file 1: Figure S1). Accordingly, all samples were subsampled to 400 reads. Subsampling eliminated 34% of all samples (22 out of 64 samples) including 9 out of 11 adenoid biopsy samples from the contaminant-filtered data (due to low sequencing depth). Consequently, adenoid biopsy data were not used in the calculation of diversity estimates for the comparison of filtered and non-filtered sequences.

Diversity estimates were performed before and after OTU filtering to compare its effect across sample types. Bray-Curtis (BC) similarity matrices were created using QIIME for principle coordinates analysis (PCoA) and PRIMER v.6 (PRIMER-E Ltd, Plymouth, UK) for SIMilarity of PERcentages (SIMPER) and Analysis of similarity (ANOSIM) analyses. SIMPER was used to determine the contribution made by specific OTUs to the observed similarity between sample types before and after contaminant filtering. ANOSIM was also performed in PRIMER to test whether there was a statistically significant difference between the MEF and the combined upper-airway samples (NP and adenoid swabs) before and after contaminant filtering.

Mann–Whitney *U* tests or *t*-tests were used to test variation in these measures between the adenoid biopsies and swabs, depending on data distribution. The relative abundance of OTUs was plotted relative to amplicon concentration using GraphPad Prism v.6.04 (GraphPad Software Inc. California, USA) with the significance tested by Spearman’s correlation. Cytoscape v2.8.2 [26] was used to create a co-occurrence model. A worksheet with the presence or absence of each OTU observed at >1.5% relative abundance was generated, showing which sample types contained identical OTUs. This spreadsheet was then uploaded to Cytoscape to generate Figure 7.

### Consent

Written informed consent was obtained from the patient’s guardian/parent/next of kin for the publication of this report and any accompanying images.

## Additional files

Additional file 1:
**Supplementary methods.** The file contains details of surgical procedures and sample collection, DNA extraction, real-time quantitative PCR, 16S rRNA amplicon library construction and sequencing, Figures S1 to S2, and Tables S1 to S4. Additional file 2:
**filtered_contaminants.fna.** The file contains sequences corresponding to OTU identities in Table 1.

## Abbreviations

ANOSIM: analysis of similarity
BC: Bray Curtis
BLAST: basic local alignment search tool
IQR: interquartile range
MEF: middle-ear fluid
MTC: multi-template control
NCBI: national Center for Biotechnology Information
NP: nasopharyngeal
OME: otitis media with effusion
OTU: operational taxonomic unit
PCoA: principal coordinate analysis
PEAR: paired-end read merger
QIIME: quantitative insights into microbial ecology
qPCR: quantitative polymerase chain reaction
rRNA: ribosomal RNA
SIMPER: similarity of percentages
