# Supplementary material for: Deriving accurate microbiota profiles from human samples with low bacterial content through post-sequencing processing of Illumina MiSeq data
Source: Microbiome. 2015 May 5;3:19. doi: 10.1186/s40168-015-0083-8 (PMC4428251; doi:10.1186/s40168-015-0083-8)
Supplement: Additional file 1: — Supplementary methods. The file contains details of surgical procedures and sample collection, DNA extraction, real-time quantitative PCR, 16S rRNA amplicon library construction and sequencing, Figures S1 to S2, and Tables S1 to S4. [file 40168_2015_83_MOESM1_ESM.docx]

**Supplementary Methods**

Details of surgical procedure and sample collection

Children were randomised to receive adenoidectomy + myringotomy (n=5/11) or adenoidectomy + tympanostomy tubes (n=6/11) pursuant to the surgical study. All clinical specimens were collected under general anaesthesia. Intraoperative collection and processing of samples was supervised by the principle author to ensure suitability for DNA based microbiological methods.

Inclusion criteria for the surgical study were:

1. Indigenous children aged 3 - 10 years old living in remote communities, and
2. OME/AOM that has been present for ≥6 months and failed medical treatment.

The clinical criteria for the diagnosis of OME was the presence of an immobile tympanic membrane on pneumatic otoscopy, supported by an air-bone gap on audiometry (conductive hearing loss >30dB) and a Type B tympanogram.

Wax and other debris was removed from the ear canal using standard techniques. Prior to myringotomy, no sterilisation of the ear canal was undertaken (myringotomy: a small surgical incision in the tympanic membrane). Following myringotomy, a suction catheter removed the MEF through the myringotomy. The effusion was collected into an *Argyle Specimen Trap* (Covidien, Massachusetts USA) by aspiration of 2ml of saline through the suction catheter. Nasopharyngeal swabs were collected according to the technique recommended by World Health Organisation Pneumococcal Working Group [1]. A sterile *Paediatric FLOQ Swab* (Copan, California USA) was passed along the floor of the nasal cavity into the nasopharynx of each child, remaining in situ for 5 seconds while being rotated 180 degrees to saturate the swab. The swab was removed and placed into a skim milk tryptone glucose glycerol broth (STGGB). Adenoidectomy was then undertaken using a curette technique. All specimens were stored on ice until the end of the surgical procedure, and then transferred to a -70 C freezer. 11 adenoid swabs were taken from the adenoid biopsies prior to DNA extraction. In addition to the 22 MEFs, 11 NP swabs, 11 adenoid biopsies and 11 adenoid swabs (total 55 samples), a number of replicate MEF samples were collected for 16S rRNA sequencing. A total of 8 swabs of suction tubing were included in the clinical samples. Additionally, for all MEF samples, 0.25ml of the 2ml solution was transferred into an STGGB tube. 10/22 MEF samples in STGGB (bilateral samples from 5 children) were therefore included in the clinical samples. Accordingly, 73 clinical samples were sent for DNA extraction. The 18 samples comprising of MEF replicates (8 MEF Tube and 10 STGGB samples) were only used for the purpose of establishing correlation plots to identify contaminant OTUs and were not used for any further downstream analysis.

DNA extraction from clinical samples

All samples were thawed on ice. After thawing, a swab of the adenoid tissue surface was prepared. The swab was then placed into 1 mL of sterile STGGB media and vortexed at full speed for 1 minute. DNA was then extracted from 200 µL of MEF, 400 µL of NP swab media, 1mL of adenoid swab media and 8-30 mg of adenoid tissue.

For MEF and swab media specimens, cellular material was pelleted by centrifugation at 7400 x g at 4ºC for 3 mins. Pellets were then resuspended in 600 μL lysis buffer consisting of Qiagen Buffer AL (Qiagen, Victoria, Australia), 2.9 mg ml^-1^ lysozyme, 0.075 mg ml^-1^ mutanolysin and 0.019 mg ml^-1^ lysostaphin. The mixture was then incubated at 37°C for 30 min.

Adenoid tissue samples were homogenised using a micropestle after addition of 50 µL of lysis buffer. Following homogenisation, a further 550 µl of lysis buffer was added and the sample mixed by pipetting. The mixture was then incubated at 37°C for 30 min.

All samples were then mechanically lysed using a FastPrep instrument (MP Biomedicals, CA, USA) with Lysing Matrix B (MP Biomedicals, CA, USA). Bead-beating was performed for 30 sec at 6.0 m sec^-1^. The supernatant was then taken and 45 µl proteinase K (20 mg ml^-1^) was added. The samples were then incubated at 65°C for 10 min. Subsequently, 200 μL of ethanol was added and the genomic DNA was purified using a QIAamp DNA Mini Kit (Qiagen), as per the manufacturer’s tissue protocol (QIAamp Micro Handbook, version 08/2003). DNA concentration and purity were measured using a NanoDrop spectrophotometer (Thermo Fisher Scientific, Australia). Samples with DNA concentrations below the dynamic range of the NanoDrop spectrophotometer were analysed using Qubit High-Sensitivity (HS) dsDNA Assay kit (Life Technologies, Australia).

Real-time quantitative PCR

Quantitative PCR to estimate the total bacterial load was performed using the primers of Nadkarni *et al*. [3] which amplify a 466-bp region between positions 331-797 of the 16S rRNA gene, based on *E. coli* numbering. The assay was performed as described previously [2]. Each 10µL qPCR reaction included 1X SensiMix^TM^ SYBR^®^ reagent (Bioline), 300nM of each primer and 1µL of template DNA. The qPCR was performed using a Rotor-Gene 6000 real-time thermocycler. The reaction conditions were an initial hold at 50ºC for 2 min followed by incubation at 95ºC for 10 min then 35 cycles of 95ºC for 15 s, 58ºC for 15 s and 72ºC for 45 s. Melt-curve analysis was then done between 80ºC-90ºC with 0.1ºC steps. As multiple amplicons form during universal 16S rRNA gene amplification, it was not possible to define a single melt-curve dissociation temperature that could be used to differentiate specific from non-specific amplicons. To overcome this limitation, the entire melt-curve was considered. Replicate analyses with irreproducible melt-curves were considered indicative of non-specific amplification. Genomic DNA from the *S. pneumoniae* ATCC49619 reference isolate was used to prepare the standard curve (1:10 serial dilution from 2000ng-200fg). Thus, the assay estimated total bacterial load assuming four ribosomal operons per cell [3]. The total bacterial load qPCR limit of detection was 90 cells based on an *S. pneumoniae* genome size of ~2Mb [GenBank:AE005672]. qPCR raw data and standard curves were prepared using the Rotor-Gene 6000 software (Corbett Research; version 1.7).

16S rRNA amplicon library construction and sequencing

Barcoded amplicon libraries for the bacterial community analysis on the Illumina MiSeq platform were generated using degenerate primers targeting the V1 and V3 hypervariable region of the bacterial 16S rRNA gene and Nextera XT index kit (Illumina, Inc., Victoria, Australia).

Amplicons were generated using fusion degenerate primers 27F (5'-TCGTCGGCAGCGTCAGATGTGTATAAGAGACAGAGRGTTTGATCMTGGCTCAG-3') and 519R (5'-GTCTCGTGGGCTCGGAGATGTGTATAAGAGACAGGTNTTACNGCGGCKGCTG-3') with ligated overhang Illumina adapter consensus sequences as shown in underlines. Initially, PCR reactions were performed on a Veriti 96-well Thermal Cycler (Life Technologies, Australia) with 12.5 µl of 2X KAPA HiFi Hotstart Ready Mix (KAPA Biosystems, MA, USA), 12.5 ng of total DNA template, 0.5 µM of each primer and nuclease-free water to a total volume of 25 µl. Samples with low DNA yield (less than 2 ng µl^-1^, as measured from the Qubit HS dsDNA assay kit) were carried out in a 50 µl PCR reaction to achieve a starting DNA amount of 12.5 ng. The PCR reactions were performed in the following program; initiation enzyme activation at 95°C for 3 min, followed by 25 cycles consisting of denaturation at 95°C for 30 sec, annealing at 55°C for 30 sec, and extension at 72°C for 30 sec. After 25 cycles, the reaction was completed with a final extension of 7 min at 72 °C. The 640 bp 16S amplicons were purified using 20 µl Agencourt Ampure XP magnetic beads (Beckman Coulter, Inc., New South Wales, Australia) according to the manufacturer's instructions, with exceptions for the 50 µl PCR reaction, where 40 µl of the bead solution was added to the sample to maintain the final polyethylene glycol concentration. The 16S amplicons were eluted from the magnetic beads in 52.5 µl of 10 mM Tris-HCl, pH8.0.

The Illumina Nextera XT Index kit with dual 8 bases indices were used to allow for multiplexing. Two unique indices located on either end of the amplicon were chosen based on the Nextera dual-indexing strategy. To incorporate the indices to the 16S amplicons, PCR reactions containing 25 µl of KAPA HiFi HotStart Ready Mix, 5 µl of each i5 and i7 index (Illumina), 5 µl of purified amplicons and nuclease-free water were mixed to a total volume of 50 µl, and were performed on a Veriti 96-well Thermal Cycler (Life Technologies). Cycling conditions consist of one cycle of 95°C for 3 min, followed by eight cycles of 95°C for 30 sec, 55°C for 30 sec, and 72°C for 30 sec, followed by a final extension cycle of 72°C for 5 min. The barcoded amplicons were then purified using 56 µl of Agencourt Ampure XT magnetic beads (Beckman Coulter, Inc.) according to the instructions of the manufacturer. The barcoded libraries were eluted from the magnetic beads in 27.5 µl of 10 mM Tris-HCl, pH8.0. Following library preparation, samples with low amplicon concentration (<2ng/µl) were excluded from sequencing.

Prior to library pooling, the barcoded libraries were quantified using Qubit HS dsDNA assay kit (Life Technologies). Libraries were mixed in approximately equal concentrations to ensure an even representation of reads per sample. The size of the pooled libraries were verified using Agilent DNA 1000 Analysis kit (Agilent Technologies, Inc., CA, USA) on the Agilent 2100 Bioanalyzer system (Agilent Technologies). The 670 bps barcoded libraries were denatured at 4 nM before diluting to a final concentration of 8 pM. The libraries were then spiked with 20% PhiX control (Illumina), and were sequenced by 2 x 300 bp paired-end sequencing on the MiSeq platform using MiSeq v3 Reagent Kit (Illumina) at the Flinders Genomics Facility, Adelaide, Australia.

**References**

1. Satzke C, Turner P, Virolainen-Julkunen A, Adrian PV, Antonio M, Hare KM, Henao-Restrepo AM, Leach AJ, Klugman KP, Porter BD, Sá-Leão R, Scott JA, Nohynek H, O'Brien KL; WHO Pneumococcal Carriage Working Group: **Standard method for detecting upper respiratory carriage of Streptococcus pneumoniae: updated recommendations from the World Health Organization Pneumococcal Carriage Working Group.** *Vaccine* 2013, **32:**165-79.
2. Marsh RL, Binks MJ, Beissbarth J, Christensen P, Morris PS, Leach AJ, Smith-Vaughan HC: **Quantitative PCR of ear discharge from indigenous Australian children with acute otitis media with perforation supports a role for *Alloiococcus otitidis* as a secondary pathogen.** *BMC Ear, Nose and Throat Disord* 2012, **12:**11.
3. Nadkarni MA, Martin FE, Jacques NA, Hunter N: **Determination of bacterial load by real-time PCR using a broad-range (universal) probe and primers set.** *Microbiol* 2002, **148:**257-266.
4. Smith-Vaughan H, Byun R, Nadkarni M, Jacques NA, Hunter N, Halpin S, Morris PS, Leach AJ: **Measuring nasal bacterial load and its association with otitis media.** BMC Ear Nose and Throat Disord 2006, **10:**10.
5. Binks M, Cheng A, Smith-Vaughan H, Sloots T, Nissen M, Whiley D, McDonnell J, Leach A: **Viral-bacterial co-infection in Australian Indigenous children with acute otitis media.** BMC Infect Dis 2011, **11:**161.​


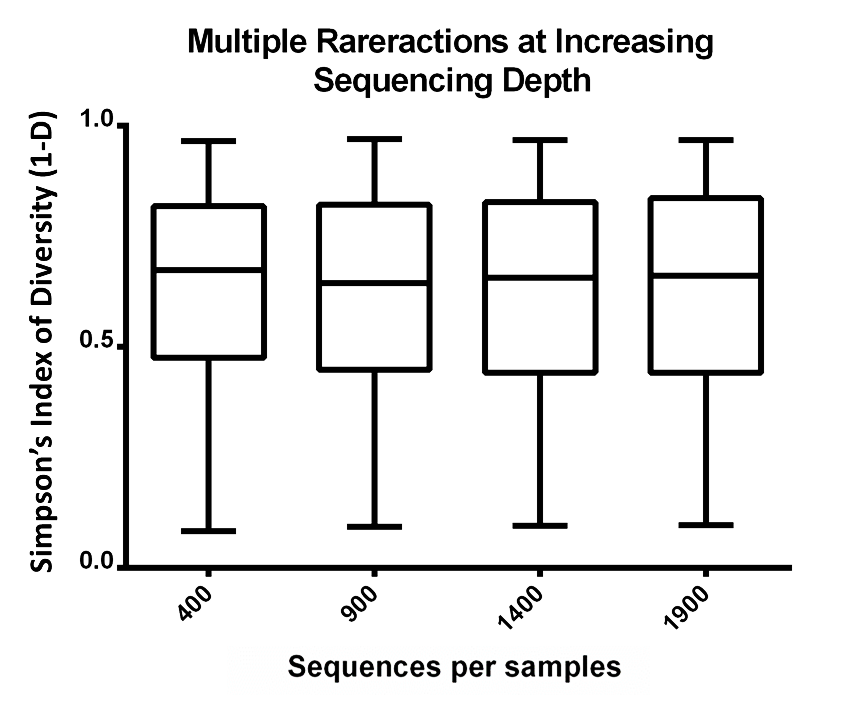


**Figure S1.** **Effect of subsampling on microbiota diversity.** Box and whisker plot of Simpson’s Index of Diversity (1-D) at four levels of subsampling. Diversity levels are shown for the 21 samples that had ≥ 2000 reads, post-filtering.


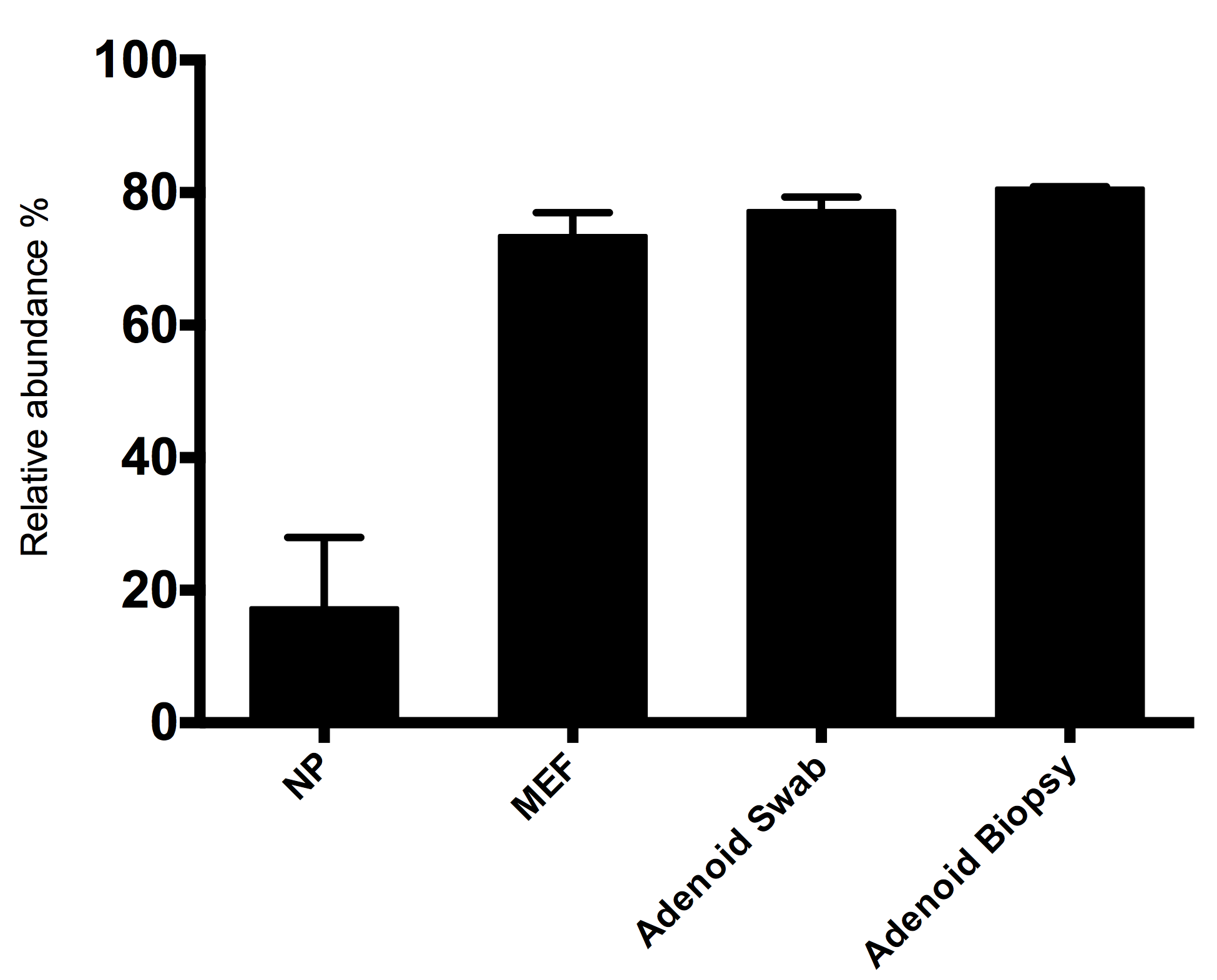


**Figure S2.** **Host-read contamination.** Bar plot of median relative abundance of host reads (Human Genome GRcH38) in each sample type. Error bars represent interquartile range.

**Table S1. Composition of multi-template control.**

* Relative abundance as a percentage of 16S rRNA gene copy number.

| Species | Strain ID | Relative abundance* |
| --- | --- | --- |
| *Alloiococcus otitidis* | ATCC51267 | 0.43 |
| *Bacteroides fragilis* | ATCC23748 | 10.02 |
| *Escherichia coli* | ATCC25922 | 0.67 |
| *Haemophilus influenzae* | ATCC49247 | 15.2 |
| *Haemophilus parainfluenzae* | ATCC7901 | 1.88 |
| *Klebsiella pneumoniae* | MSHR110128 | 37.62 |
| *Moraxella catarrhalis* | ATCC8176 | 10.39 |
| *Neisseria meningitidis* | ATCC13090 | 7.39 |
| *Proteus mirabilis* | ATCC12453 | 0.39 |
| *Pseudomonas aeruginosa* | ATCC27853 | 2.97 |
| *Staphylococcus aureus* | ATCC25923 | 5.64 |
| *Streptococcus mitis* | ATCC49619 | 0.05 |
| *Streptococcus pneumoniae* | ATCC6249 | 7.36 |

**Table S2. Composition of multi-template control, used in the assessment of reference databases.**

| Expected | |  |  |
| --- | --- | --- | --- |
| Family | **Expected** | **Genus** | **Expected** |
| Enterobacteriaceae | 44.36% | *Klebsiella* | 43.36% |
| Bacteroidaceae | 13.63% | *Bacteroides* | 13.63% |
| Moraxellaceae | 7.54% | *Moraxella* | 7.54% |
| Pseudomonadaceae | 7.24% | *Pseudomonas* | 7.24% |
| Pasteurellaceae | 8.22% | *Haemophilus* | 8.22% |
| Streptococcaceae | 6.20% | *Streptococcus* | 6.20% |
| Staphylococcaceae | 6.19% | *Staphylococcus* | 6.19% |
| Neisseriaceae | 6.19% | *Neisseria* | 6.19% |
| Carnobacteriaceae | 0.29% | *Escherichia* | 0.71% |
|  |  | *Proteus* | 0.36% |
|  |  | *Alloiococcus* | 0.29% |

**Table S3. Analysis of multi-template control sequence data through comparison with Greengenes reference database.**

* = BLAST identity at >95%.

| GREENGENES (OTUs with relative abundance >.40%) | | | | | |  |
| --- | --- | --- | --- | --- | --- | --- |
| #OTU ID | **Seq.** | **Rel. Abund.** | **Family** | **Genus** | **Is the highest classification assigned by Greengenes correct according to NCBI?** | |
| 183603 | 14426 | 27.22% | Bacteroidaceae | *Bacteroides* | Yes | |
| N.R.OTU0 | 9093 | 17.16% | Unassigned | Unassigned | not assigned (*Moraxella*)* | |
| N.R.OTU1 | 8120 | 15.32% | Pasteurellaceae | *Haemophilus* | Yes | |
| 197286 | 6936 | 13.09% | Enterobacteriaceae | Unassigned | Yes | |
| 4365567 | 3254 | 6.14% | Neisseriaceae | *Neisseria* | Yes | |
| 222184 | 2167 | 4.09% | Pseudomonadaceae | *Pseudomonas* | Yes | |
| 4376233 | 667 | 1.26% | Enterobacteriaceae | Unassigned | Yes | |
| 4305793 | 637 | 1.20% | Pasteurellaceae | *Haemophilus* | Yes | |
| 4455250 | 482 | 0.91% | Streptococcaceae | *Streptococcus* | Yes | |
| N.C.R.OTU1333 | 418 | 0.79% | Unassigned | Unassigned | not assigned (*Haemophilus*)* | |
| 560629 | 329 | 0.62% | Enterobacteriaceae | *Proteus* | Yes | |
| N.C.R.OTU356 | 289 | 0.55% | Unassigned | Unassigned | not assigned (*Moraxella*)* | |
| N.C.R.OTU1974 | 253 | 0.48% | Unassigned | Unassigned | not assigned (*Moraxella*)* | |
| N.C.R.OTU3450 | 235 | 0.44% | Unassigned | Unassigned | not assigned (*Neisseria*)* | |
| N.C.R.OTU6077 | 220 | 0.42% | Unassigned | Unassigned | not assigned (*Staphylococcus*)* | |
| N.C.R.OTU1205 | 219 | 0.41% | Unassigned | Unassigned | not assigned (*Moraxella*)* | |
| N.C.R.OTU231 | 212 | 0.40% | Unassigned | Unassigned | not assigned (*Moraxella*)* | |

**Table S4.** **Analysis of multi-template control sequence data through comparison with SILVA reference database.**

* = NCBI BLAST identity at >95%.

| SILVA (OTUs with relative abundance >.40%) | | | | | |  |
| --- | --- | --- | --- | --- | --- | --- |
| #OTU ID | **Seq.** | **Rel. Abund.** | **Family (unless otherwise specified)** | **Genus** | **Is the highest classification assigned by SILVA correct according to NCBI?** | |
| DQ807326 | 14425 | 26.71% | Bacteroidales (order) | Unassigned | Yes | |
| N.R.OTU4 | 8993 | 16.65% | Moraxellaceae | *Moraxella* | Yes | |
| N.R.OTU0 | 8827 | 16.35% | Pasteurellaceae | *Haemophilus* | Yes | |
| EU775630 | 4783 | 8.86% | Enterobacteriaceae | Unassigned | Yes | |
| GP740200 | 3806 | 7.05% | Neisseriaceae | *Neisseria* | Yes | |
| GQ258634 | 2155 | 3.99% | Pseudomonadaceae | *Pseudomonas* | Yes | |
| EU775715 | 2059 | 3.81% | Enterobacteriaceae | Unassigned | Yes | |
| FJ558078 | 614 | 1.14% | Streptococcaceae | *Streptococcus* | Yes | |
| EU775611 | 576 | 1.07% | Enterobacteriaceae | Unassigned | Yes | |
| N.R.OTU11 | 399 | 0.74% | Pasteurellaceae | *Haemophilus* | Yes | |
| N.R.OTU10 | 352 | 0.65% | Unassigned | Unassigned | not assigned (*Moraxella*)* | |
| HQ407310 | 340 | 0.63% | Enterobacteriaceae | *Proteus* | Yes | |
| N.R.OTU16 | 321 | 0.59% | Unassigned | Unassigned | not assigned (*Moraxella*)* | |
| N.R.OTU8 | 262 | 0.49% | Unassigned | Unassigned | not assigned (*Moraxella*)* | |
| N.R.OTU7 | 230 | 0.43% | Unassigned | Unassigned | not assigned (*Neisseria*)* | |
| N.C.R.OTU1223 | 219 | 0.41% | Unassigned | Unassigned | not assigned (*Moraxella*)* | |
| N.C.R.OTU6242 | 218 | 0.40% | Staphylococcaceae | *Staphylococcus* | Yes | |
